# Influence of Family Processes on Internet Addiction Among Late Adolescents in Hong Kong

**DOI:** 10.3389/fpsyt.2019.00113

**Published:** 2019-03-12

**Authors:** Daniel T. L. Shek, Xiaoqin Zhu, Diya Dou

**Affiliations:** Department of Applied Social Sciences, The Hong Kong Polytechnic University, Hong Kong, China

**Keywords:** adolescent, Chinese students, internet addiction, father, mother, growth curve modeling

## Abstract

The present study investigated how the quality of the parent–child subsystem (indexed by behavioral control, psychological control, and parent–child relationship) predicted Internet addiction (IA) levels and change rates among senior high school students. It also examined the concurrent and longitudinal influence of the father- and mother-related factors on adolescent IA. At the beginning of the 2009/2010 school year, we randomly selected 28 high schools in Hong Kong and invited Grade 7 students to complete a questionnaire annually across the high school years. The present study used data collected in the senior high school years (Wave 4–6), which included a matched sample of 3,074 students (aged 15.57 ± 0.74 years at Wave 4). Growth curve modeling analyses revealed a slight decreasing trend in adolescent IA in senior high school years. While higher paternal behavioral control predicted children's lower initial level of and a slower drop in IA, maternal behavioral control was not a significant predictor of these measures. In contrast, higher maternal but not paternal psychological control showed a significant relationship with a higher initial level of and a faster drop in adolescent IA. Finally, better father–child and mother–child relationships predicted a lower initial level of IA among adolescents. However, while a poorer mother–child relationship predicted a faster decline in adolescent IA, father–child relationship quality did not. With the inclusion of all parent–child subsystem factors in the regression analyses, paternal behavioral control and maternal psychological control were identified as the two unique concurrent and longitudinal predictors of adolescent IA. The present findings delineate the essential role of parental control and the parent–child relationship in shaping children's IA across senior high school years, which is inadequately covered in the scientific literature. The study also clarifies the relative contribution of different processes related to the father–child and mother–child subsystems. These findings highlight the need to differentiate the following: (a) levels of and rates of change in adolescent IA, (b) different family processes in the parent–child subsystem, and (c) father- and mother-related factors' contribution to adolescent IA.

## Introduction

The use of the Internet has grown rapidly in the past two decades, especially amongst the adolescent population. As the Internet becomes pervasive and increasingly indispensable in young individuals' lives, Internet addiction (IA) has also emerged as a serious public health issue due to its close relationships with adolescent health problems, risk behaviors, and social functioning problems (1–3).

Preventing IA requires efforts taken in various sectors such as family, school, and other social institutions. Family factors, such as positive family functioning, parental monitoring, and healthy parental–child relationship, play a vital role in this process (4–6). Among different parenting characteristics, parental control was intimately related to problematic adolescent behaviors [e.g., (7, 8)].

Parental control includes behavioral and psychological control (9). Behavioral control pertains to the use of disciplinary strategies and supervisory functions to regulate children's behavior, while psychological control attempts to shape children's behaviors through strategies like guilt or anxiety induction, and love withdrawal (10). Research findings have consistently shown that the behavioral control of parents is positively related to favorable developmental outcomes in children, while psychological control harms adolescent health (7, 8, 11). As Barber et al. (11) concluded, higher parental behavioral control is related to higher levels of adolescent competence, self-discipline, and school performance as well as lower levels of problematic behaviors. When parents provide clear regulations regarding children's Internet use or monitor their Internet use in an appropriate way, adolescents have a lower chance to show symptoms of IA. In contrast, parental psychological control tends to harm children's self-esteem, and increase developmental problems and maladjustment, because psychological control hurts adolescents' emotional functioning and the sense of self (12). For example, in a sample of 5,806 seventh graders, Wang et al. (8) found that the psychological control of parents predicted students' dampened emotional functioning in China and the United States.

There are three issues that should be addressed when examining the influence of parents on adolescent developmental outcomes. First, while parental control has been identified as an important determinant of adolescent development, the impact of the parent–child relationship has not been adequately examined. According to Shek (13), adolescents' satisfaction with their parents' control and their willingness to communicate with their parents are important relational qualities that should be considered when examining adolescent adjustment. Primarily, children's satisfaction with their parents' control reflects the quality of the parent-child relationship. Whether adolescents themselves regard parental control as reasonable or not is an important factor to be considered when understanding the influence of parental behavioral and psychological control. Pomerantz and Eaton (14) argued that some adolescents might regard parental control as an expression of care. Hence, these adolescents may gain more positive influence from parental control. This argument was supported by Kakihara (15), who found that adolescents interpreted strong parental behavioral control in specific domains as indicative of competence or intrusiveness. Thus, Kakihara drew attention to the possibility of multiple yet “contradictory” interpretations of parenting reported by adolescents. Hence, it is important to consider the quality of parent–child relationship when examining the impact of parental control.

Besides, parent–adolescent communication also influences adolescent development. Research showed that healthy parent–adolescent communication provides a safe environment for adolescents to comfortably disclose themselves (16), while problematic parent–adolescent communication is often associated with increased adolescent risk behaviors (17). Forehand et al. (18) found that families of adolescents with behavior problems experienced disagreements more frequently and had less supportive parent–child relationships than their counterparts did. Cottrell et al. (19) revealed that open parent–child communication was positively associated with parental monitoring. However, as Burk and Laursen (20) pointed out, little is known about how specific relationship attributes contribute to specific developmental problems such as IA.

The second issue concerns the differential influence of fathers and mothers on adolescent development. Historically, there has been an absence of parenting research examining the relationships between adolescents and their fathers (21). In addition, instead of separating paternal and maternal impacts, many studies just considered overall parenting characteristics (i.e., adolescent perception of their parents), which hinders the understanding of the complex dynamics in this process (22). Some recent studies have recognized the distinction between maternal and paternal factors on adolescent developmental outcomes, but the majority adopted a single perspective focusing on either mother' or fathers' influence. For example, Leung et al. (23) explored maternal control in a sample of 432 poor Chinese single-mother families. Studies on paternal involvement were reviewed in Pleck and Masciadrelli's (24) research, which showed that paternal involvement was related to positive child development. However, to have a holistic picture of how family factors systematically influence adolescent IA, it is important to include both maternal and paternal factors. Hence, it is argued that when examining parental impacts, both maternal and paternal factors should be taken into account.

However, the existing research findings regarding the differential effects of fathers and mothers are equivocal. For example, Giles and Price (25) found that only maternal psychological control positively predicted problematic computer use in children. In contrast, Lansford et al. (26) revealed that only paternal psychological control accounted for specific variance in children's developmental problems. The authors reported a similar result on the unique predictive effect of paternal knowledge on boys' externalizing problems. In Xu's research (2) involving 5,122 Chinese adolescents, results showed that compared to the father–child relationship, the mother–child relationship had a stronger association with children's IA. While these studies illustrated the differences between maternal and paternal impacts, the findings were inconclusive. Furthermore, they generally focused on a single parenting factor (e.g., psychological control) rather than simultaneously considering different aspects of the parent–child subsystem.

The third issue pertains to research design, in that most of the existing findings in this field are based on cross-sectional studies. Few studies have used longitudinal data to examine the growth rate of IA related to different family factors (4, 6). For example, Wang et al. (17) research using three-year longitudinal data on 913 Bahamian students revealed that parental control during early adolescence predicted a decrease in risk behavior in middle adolescence. Yu and Shek's (27) longitudinal research on students in Hong Kong revealed that good family functioning predicted a lower probability of having IA. Regarding the impacts of parental factors on the growth rate of IA, Shek et al. (28) study involving 3,328 Chinese students indicated that stronger paternal behavioral control was associated with a slower drop in children's IA, and stronger maternal psychological control was linked to a faster decrease in children's IA. Although their findings filled the knowledge gaps in the field, the study only covered early adolescence. More efforts should be made to examine the differences between maternal and paternal parenting styles among late adolescents (29).

In short, longitudinal research focusing on senior high school students' IA is urgently needed to understand how the parent–child relationship qualities shape adolescent IA. First, the dynamics of parenting and the family relationship functioning of senior high school students of IA are different from that of junior students. Students in late adolescence are physically and psychologically mature, demanding more freedom and flexibility. Parental control may not lead to the desired effect to adolescents at this stage. For example, Rogers et al. (30) suggested a stronger association between parental psychological control and internalizing problems for senior high school students than for junior students, because older adolescents might have stronger need for autonomy and more diverse internalizing and externalizing behaviors. Second, as the patterns in which mother- and father-related factors influence adolescents may evolve differently as both parents and adolescents age, longitudinal research would enhance our understanding of the differences between the influence of maternal and paternal factors on adolescent IA.

## The Present Study

To fill these three research gaps, the current study investigated how parent–child subsystem factors influence the levels and rates of change in adolescent IA over senior high school years. Specifically, the study addressed the following three research questions. The first question is as follows: “do parental control (behavioral and psychological) and father– and mother–child relationship qualities predict children's initial level of IA at the beginning of senior high school life?” Drawing from extant literature (7, 8, 11) and related findings in early adolescence (28), it was expected that higher paternal and maternal behavioral control would predict a lower initial level of adolescent IA in senior high school years (Hypotheses 1a and 1b), while higher paternal and maternal psychological control would be associated with a higher initial level of adolescent IA (Hypotheses 1c and 1d). Regarding the parent–child relationship, we hypothesized that better relationships between the child and both parents would predict a lower initial level of IA (Hypotheses 1e and 1f).

The second research question is as follows: “how are parent–child subsystem qualities related to the developmental trajectory of adolescent IA across senior high school years?” In this field, only one previous study found that paternal behavioral control, maternal psychological control, as well as relationships between the child and both parents exerted significant predictive effects on the rate of change in IA during junior high school years (28). However, the related findings were at odds with the general expectations that favorable parental factors would be associated with a faster drop in IA as an indicator of positive adjustment in the long run. Given that only limited evidence is available, we still formed the present hypotheses based on the general theoretical expectations that positive parenting and a better parent–child relationship would be related to children's positive development (31). Specifically, it was expected that higher behavioral control of fathers (Hypothesis 2a) and mothers (Hypothesis 2b), as well as better father–child (Hypothesis 2c) and mother–child relationships (Hypothesis 2d), would predict a faster decline in children's IA because these factors were considered as positive aspects of parental impacts. Additionally, as a form of negative parenting, higher paternal and maternal psychological control were expected to predict a slower decrease in adolescent IA (Hypotheses 2e and 2f, respectively).

The third question is as follows: “what is the concurrent and longitudinal influence of paternal and maternal factors on adolescent IA during senior high school years?” While there is support for the relatively stronger influence of paternal parenting (26), other findings suggest a stronger influence for maternal parenting (2, 25). Besides the inconclusive picture, very few studies have distinguished different aspects of parenting practices (e.g., behavioral and psychological control and the relationship between parents and children) and examined the long-term impact using longitudinal data (28). Due to these factors, the following two general competing hypotheses were advanced: fathers are more influential than mothers in influencing adolescent IA (Hypothesis 3a), and mothers are more influential than fathers in influencing adolescent IA (Hypothesis 3b). All the above hypotheses are summarized in Table 1.

**Table 1:** Summary of hypotheses and findings of the present study.

**Research question**	**Hypotheses**	**Brief descriptions**	**Findings**		
			**Full sample**	**Male sample**	**Female sample**
NA		A decline trend of adolescent IA over time	Yes	Yes	Yes
One	1a	Paternal behavioral control negatively predicts the initial level of adolescent Internet addiction (IA)	Yes	Yes	Yes
	1b	Maternal behavioral control negatively predicts the initial level of adolescent IA	No	No	No
	1c	Paternal psychological control positively predicts the initial level of adolescent IA	No	No	No
	1d	Maternal psychological control positively predicts the initial level of adolescent IA	Yes	Yes	Yes
	1e	Father–child relationship quality negatively predicts the initial level of adolescent IA	Yes	Yes	Yes
	1f	Mother–child relationship quality negatively predicts the initial level of adolescent IA	Yes	Yes	No
Two	2a	Higher paternal behavioral control will predict a faster decline in adolescent IA	No (opposite direction)	No (opposite direction)	No (opposite direction)
	2b	Higher maternal behavioral control will predict a faster decline in adolescent IA	No	No (opposite direction)	No
	2c	Better father–child relationship quality will predict a faster decline in adolescent IA	No	No	No
	2d	Better mother–child relationship quality will predict a faster decline in adolescent IA	No (opposite direction)	No (opposite direction)	No
	2e	Higher paternal psychological control will predict a slower decrease in adolescent IA	No	No	No
	2f	Higher maternal psychological control will predict a slower decrease in adolescent IA	No (opposite direction)	No (opposite direction)	No
Three	3a	Paternal factors are more influential than maternal factors in shaping adolescent IA	Yes	Yes	Yes
	3b	Maternal factors are more influential than paternal factors in influencing adolescent IA	No	No	No

*Opposite direction indicates the a significant but opposite predictive effect compared to hypothesized effect*.

The present study also considered important demographic characteristics including student gender, family economic status, and family intactness. Specifically, boys often report a higher level of Internet use and a higher interest in online games than girls do (4). Some studies found that parenting effects vary among boys and girls. For example, Shek (32) examined parenting functions among Chinese adolescents with disadvantageous background and found that paternal parenting exerted more influence on boys' mental health and problem behavior, while maternal parenting played a major role in affecting girls' mental health and problem behavior. In contrast, Rogers et al. (30) reported that fathers' psychological control was more influential on daughters than on sons regarding externalizing behavior. Shi et al. (4) examined the relation between family functioning and children's IA and found that the path from family function to IA (via loneliness) was significant only for girls. By including student gender, this study attempted to contribute to the ongoing discussion about differential parenting influence on IA amongst boys and girls.

## Materials and Methods

### Participants and Procedures

This study was derived from a 6-year longitudinal project that investigated the adjustment of Chinese adolescents in Hong Kong. A cluster sampling method was used. First, based on a school list provided by the Educational Bureau in Hong Kong, we formed a list of candidate schools including 399 government-funded or aided Chinese-speaking secondary schools in different districts of Hong Kong. Second, 30 schools were randomly selected and invited to join the project. If the selected school rejected our invitation, we invited an alternate school randomly selected from the candidate schools in the same district. Eventually, 28 schools agreed to join the project (Hong Kong Island: 5 schools; Kowloon: 7 schools; New Territories: 16 schools). Third, at the beginning of the 2009/2010 academic year, all the students in the first year of high school study (i.e., Grade 7) in the 28 participating schools were invited to complete a questionnaire and were followed up annually through the high school years, resulting in a 6-wave data set.

In 2009/2010, the number of Grade 7 students in the participating schools (*N* = 4,531) accounted for 7.12% of the total number of Grade 7 students in all the candidate schools (*N* = 63,620). At Wave 1 data collection, 3,328 students completed the questionnaire, suggesting a response rate of 73.45%. As demographic information of non-respondents who did not participate in any wave of data collection was not available, we were not able to compare participating students with non-respondents regarding their background characteristics. However, according to Shek et al. (33), the sample attributes of the project were similar to the demographic profile of the general adolescent population in secondary schools in Hong Kong.

Ethical approval for this project was obtained from the Human Subjects Ethics Sub-committee (HSESC) (or its Delegate) at The Hong Kong Polytechnic University. All involved parties, including participating schools, student participants, and their parents, provided informed written consent. In all occasions of data collection, trained research staff administrated the questionnaires, using a paper-and-pencil mode, in quiet classrooms at the participating schools. Administrators clearly instructed the student participants to provide honest responses based on their own interpretation of the questions.

The present study utilized Wave 4–6 data collected in the senior high school years (Grades 10–12). While data collection at Wave 5 took place 1 year (i.e., 12 months) after Wave 4, Wave 6 data collection was conducted approximately 10 months after Wave 5. This is because students at Grade 12 in Hong Kong have to concentrate on preparing for the public examination during the last few months of senior high school. The number of participants completing the questionnaire at each wave varied (Wave 4: *N* = 3,973, Wave 5: *N* = 3,683, and Wave 6: *N* = 3,498) due to students' absence at the time of data collection, transferring schools, or dropping out of schools. Among the 3,973 students who completed the survey at Wave 4, 3,397 and 3,237 completed the survey at Wave 5 and 6, respectively. From Wave 4 to Wave 5, were more participants withdrew from the study. One possible explanation is that some students might take vocational education, or they might go to work after reaching Hong Kong's legal working age of 15 years old. Across the three waves, 3,074 participants (aged 15.57 ± 0.74 years at Wave 4) were successfully matched, which included 1,577 (51.30%) boys and 1,497 (48.70%) girls. The matched sample was used in the present study.

Comparisons between the matched sample included in this study (*N* = 3,074) and who withdrew from the study after Wave 4 (i.e., *N* of dropouts = 899) showed no significant differences in family economic status and family intactness. However, the matched sample included a higher percentage of female adolescents (χ^2^_(1)_ = 10.26, *p* < 0.01, *ϕ* = 0.05). Besides, adolescents in the matched sample (aged 15.57 ± 0.74 at Wave 4) were slightly younger than the dropouts [aged 15.88 ± 0.94, *t*_(3861)_ = −9.95, *p* < 0.001, Cohen's *d* = 0.37]. Regarding the key variables considered in the current study, no significant differences were observed in IA [*t*_(3971)_ = −1.61, *p* = 0.11], paternal psychological control [*F*_(1, 3543)_ = −3.12, *p* = 0.08], and maternal behavioral control [*F*_(1, 3543)_ = −2.69, *p* = 0.10] measured at Wave 4. However, participants in the matched sample reported a slightly higher level of paternal behavioral control, better relationships with fathers and mothers, and a slightly lower level of maternal psychological control (*F* values ranged from 5.18 to 9.25, *ps* < 0.05, partial *η*^2^ ranged from 0.001 to 0.003) at Wave 4. However, the effect size was low.

### Instruments

Among the multiple measures used in the questionnaire, IA and quality of the parent–child subsystem were the key measures, and gender, participants' family economic condition, and family intactness were the control variables in the present study.

#### Internet Addiction (IA)

Kimberly Young has developed several questionnaires to assess addicted behaviors related to Internet use, including the brief 8-item questionnaire, 10-item questionnaire, and the 20-item questionnaire. For Young's 8-item IA questionnaire, items were modified from criteria for pathological gambling in the *Diagnostic and Statistical Manual of Mental Disorders—Fourth Edition* (DSM-IV) (34). The 10-item questionnaire included 10 Internet addiction symptoms (35). Shek and his collaborators translated the 10-item scale into a Chinese IA measurement, which exhibited good psychometric properties in previous research (36–38). This study utilized the 10-item Chinese IA questionnaire to assess adolescent IA. Participants responded to 10 items using a dichotomous scale (“Yes” or “No”) to indicate whether they demonstrated the listed 10 addiction behaviors related to Internet use in the past year (see Table 2). Participants' IA was indexed by the number of “yes” answers they provided in the questionnaire. In this study, the Cronbach's αs of the questionnaire ranged from 0.79 to 0.82 across the three waves (see Table 3), indicating good internal consistency.

**Table 2:** Participants' answers on Internet Addiction questionnaire across the three waves (*N* = 3,074).

**Questionnaire items**	**Wave 4 (%)**		**Wave 5 (%)**		**Wave 6 (%)**	
	**Yes**	**No**	**Yes**	**No**	**Yes**	**No**
1. Do you feel preoccupied with the Internet or on-line services and think about it while off-line?	27.3	72.7	24.3	75.7	19.6	80.4
2. Do you feel a need to spend more and more time on-line to achieve satisfaction?	21.0	79.0	17.7	82.3	15.7	84.3
3. Have you repeatedly made unsuccessful efforts to control, cut back, or stop Internet use?	18.9	81.1	17.9	82.1	17.8	82.2
4. Do you feel restless, moody, depressed, or irritable when attempting to cut down or stop Internet use?	9.6	90.4	9.3	90.7	7.7	92.3
5. Do you stay on-line longer than originally intended?	47.9	52.1	48.5	51.5	45.3	54.7
6. Have you jeopardized or risked the loss of a significant relationship, job, educational or career opportunity because of the Internet?	20.6	79.4	23.1	76.9	20.9	79.1
7. Have you lied to family members, teachers, social workers	12.4	87.6	11.7	88.3	10.7	89.3
8. Do you use the Internet as a way of escaping from problems or of relieving a dysphoric mood (e.g., feelings of helplessness, guilt, anxiety, depression)?	20.4	79.6	21.9	78.1	19.0	81.0
9. Do you keep returning even after spending too much money on online fees?	8.6	91.4	8.6	91.4	7.4	92.6
10. Do you feel depressed, irritable, moody, or anxious when you are offline?	7.1	92.9	7.5	92.5	7.3	92.7

**Table 3:** Reliability of scales and description of variables across the three waves.

**Scale**	**Number of item**	**Wave**	**Cronbach's α**	**Mean inter-item correlation**	**Range**	***M***	***SD***
Internet Addiction Test	10	Wave 4	0.79	0.29	0–10	1.94	2.22
		Wave 5	0.80	0.30	0–10	1.91	2.25
		Wave 6	0.82	0.33	0–10	1.71	2.23
Father–Child Subsystem Quality Scale	17						
Paternal behavioral control	7	Wave 4	0.89	0.53	1–4	2.48	0.59
		Wave 5	0.89	0.53	1–4	2.46	0.58
		Wave 6	0.90	0.55	1–4	2.44	0.59
Paternal psychological control	4	Wave 4	0.86	0.61	1–4	2.19	0.69
		Wave 5	0.86	0.60	1–4	2.17	0.66
		Wave 6	0.88	0.65	1–4	2.16	0.68
Father–child relational quality	6	Wave 4	0.90	0.61	1–4	2.73	0.62
		Wave 5	0.90	0.60	1–4	2.72	0.61
		Wave 6	0.90	0.62	1–4	2.71	0.60
Mother–Child Subsystem Quality Scale	17						
Maternal behavioral control	7	Wave 4	0.89	0.53	1–4	2.89	0.56
		Wave 5	0.89	0.54	1–4	2.86	0.56
		Wave 6	0.88	0.51	1–4	2.84	0.53
Maternal psychological control	4	Wave 4	0.89	0.67	1–4	2.26	0.73
		Wave 5	0.89	0.68	1–4	2.24	0.71
		Wave 6	0.91	0.71	1–4	2.23	0.72
Mother–child relational quality	6	Wave 4	0.90	0.60	1–4	2.94	0.58
		Wave 5	0.90	0.61	1–4	2.93	0.57
		Wave 6	0.90	0.60	1–4	2.93	0.55

#### Quality of the Father– and Mother–Child Subsystems

In the questionnaire, different paternal and maternal factors were measured by a reliable and validated scale entitled “Parent–Child Subsystem Quality Scale (PCSQS)” (16, 39). The PCSQS includes two 17-item subscales on paternal and maternal factors, respectively. The 17 items in each subscale can be further grouped into three dimensions. The first dimension includes seven items that assess paternal/maternal behavioral control, as indexed by each parent's expectation (e.g., “my father/mother expects me to exhibit good behavior in school”), knowledge (e.g., “my father/mother asked me about what I did after school”), and monitoring (e.g., “my father/mother actively understands my afterschool activities”). The second dimension comprises four items measuring the father's/mother's psychological control (e.g., “my father/mother often wants to change my mind or feelings about things”). The third dimension comprises six items that assess the quality of the father–/mother–child relationship, indexed by children's satisfaction with their parents' control (e.g., “my father's/mother's discipline of me is reasonable”) and children's active communication with their parents (e.g., “I share my feelings with my father/mother”). A 4-point scale (1 = “strongly disagree,” 4 = “strongly agree”) is used to rate each item. Participants' score on each dimension is indicated by the average score across all items included in it. In the present study, all subscales of the PCSQS demonstrated good reliability, as reflected by the high Cronbach's αs (0.88–0.91) across waves (see Table 3).

#### Family Economic Condition

Participants' family economic conditions were indexed by whether their family was living on welfare received from the “Comprehensive Social Security Assistance (CSSA) Scheme” of the Hong Kong Government. In the current study, the 182 (5.9%) adolescents who reported that their family was living on welfare from the CSSA at Wave 4 were grouped as “having family economic disadvantage,” and the other 2,684 (87.3%) participants whose family was not living on welfare from the CSSA at Wave 4 were categorized as “not having family economic disadvantage.”

#### Family Intactness

Parental marital status reported by the participants at Wave 4 was used to index family intactness. Specifically, if parents were in the first marriage, participants (*n* = 2,528, 82.2%) were categorized as “having an intact family.” Separation, divorce, or second marriage of parents was treated as an indicator of living in a “non-intact family” (*n* = 534, 17.4%).

### Plan of Analysis

The statistical analysis plan was the same as that used in Shek et al.'s (28) study involving early adolescents. Specifically, we first analyzed the reliability of measures, descriptions, and correlations among variables. Subsequently, individual growth curve (IGC) modeling was utilized to investigate the predictive effects of different parenting factors on the initial level of adolescent IA as well as its developmental trajectory across senior high school, to address the first two research questions. In the present IGC analysis, time (i.e., Wave 4 = 0, Wave 5 = 1, and Wave 6 = 1.83), as the Level 1 predictor, was nested into Level 2 predictors, which included both control variables and measures of the parent-child subsystem quality, leading to 2-level hierarchical models.

Testing of the hierarchical models in the present study followed procedures that have been widely adopted in previous research (28, 40–42). Basically, four models were compared. Model 1 was an unconditional mean model. Model 2 was a linear growth model that only involved Level 1 predictors (i.e., time). Model 3 was also a linear growth model, which further involved the control variables as Level 2 predictors in addition to Level 1 predictors. Model 4 further included the different aspects of the parent-child subsystem quality as Level 2 predictors in addition to Model 3 predictors. Using these procedures, any individual variability in the initial level of and the change rate of IA caused by Level 2 predictors could be identified. In this study, the three parental factors (i.e., behavioral control, psychological control, and quality of the parent-child relationship) were investigated as Level 2 predictors in Model 4a, 4b, and 4c, respectively. To explore any potential gender effect regarding parental influence on the initial level of adolescent IA and its change over time, we also tested gender-based IGC models.

Following previous studies (41, 42), we used three indices to index model fit, “−2log likelihood,” “Akaike Information Criterion” (AIC), and “Bayesian Information Criterion” (BIC). For these indices, a smaller value indicates a better model fit. Before performing IGC analyses, we dummy coded the three control variables as follows: “female” = “−1,” “male” = “1”; “having family economic disadvantage” = “−1,” “without family economic disadvantage” = “1”; “non-intact family” = “−1,” “intact family” = “1.” Meanwhile, parental factors were standardized.

The third research question was addressed by multiple regression analyses examining cross-sectional as well as longitudinal predictive effects of father–related factors, mother–related factors, and all parenting factors on adolescent IA. In short, the present study examined cross-sectional effects of parental factors on children's IA at all three waves. For longitudinal predictive effects, we examined the predictive effects of parental factors at Wave 4 on children's IA at Waves 5 and 6. In addition, to test whether children's gender would moderate parental influence on adolescent IA, we further included the interactions between gender and each factor related to parent–child subsystem quality in regression analyses.

## Results

### Correlations Among Variables

Table 4 shows the correlation coefficients among the variables examined in the present study. While parental behavioral control and relationships between parents and children were negatively associated with children's IA, there were positive correlations between parental psychological control and children's IA. These results support the general expectations. In addition, compared to female adolescents, male adolescents demonstrated a higher level of IA across all waves.

**Table 4:** Correlations among variables.

	**Variables**	**1**	**2**	**3**	**4**	**5**	**6**	**7**	**8**	**9**	**10**	**11**
1.	Gender^a^	–										
2.	FES^b^	0.05*	–									
3.	FI ^c^	0.04*	0.30***	–								
4.	W4 PBC	0.01	0.10***	0.14***	–							
5.	W4 PPC	0.13***	0.03	0.05**	0.13***	–						
6.	W4 FCRQ	−0.03	0.08***	0.17***	0.66***	−0.17**	–					
7.	W4 MBC	−0.10***	0.07***	0.10***	0.43***	0.03	0.33***	–				
8.	W4 MPC	0.09***	0.01	−0.02	0.03	0.45***	−0.10***	0.06***	–			
9.	W4 MCRQ	−0.11***	0.03	0.08***	0.31***	−0.06**	0.40***	0.63***	−0.26***	–		
10.	W4 IA	0.11**	−0.04*	−0.03	−0.16***	0.09***	−0.14***	−0.10***	0.13***	−0.13***	–	
11.	W5 IA	0.05*	−0.05**	−0.03	−0.11***	0.06**	−0.11***	−0.06**	0.10***	−0.07***	0.60***	–
12.	W6 IA	0.05**	−0.03	−0.03	−0.09***	0.08***	−0.09***	−0.05**	0.09***	−0.06***	0.52***	0.61***

*The correlational patterns between parent-child subsystem qualities at different waves and other variables were the same, so only the results on Wave 4 parenting characteristics were presented in the table due to space limit. FES, Family economic status; FI, Family intactness; PBC, Paternal behavioral control; PPC, Paternal psychological control; FCRQ, Father–child relational quality; MBC, Maternal behavioral control; MPC, Maternal psychological control; MCRQ, Mother–child relational quality; IA, Internet addiction; W4, Wave 4; W5, Wave 5; W6, Wave 6*.

a *Female = −1, Male = 1*.

b *Having economic disadvantage = −1, Not having economic disadvantage = 1*.

c *Non-intact family = −1, Intact family = 1*.

* *p < 0.05*;

** *p < 0.01*;

*** *p < 0.001*.

### Developmental Trajectory of Adolescent IA and Predictive Effects of Control Variables

**Figure 1:**
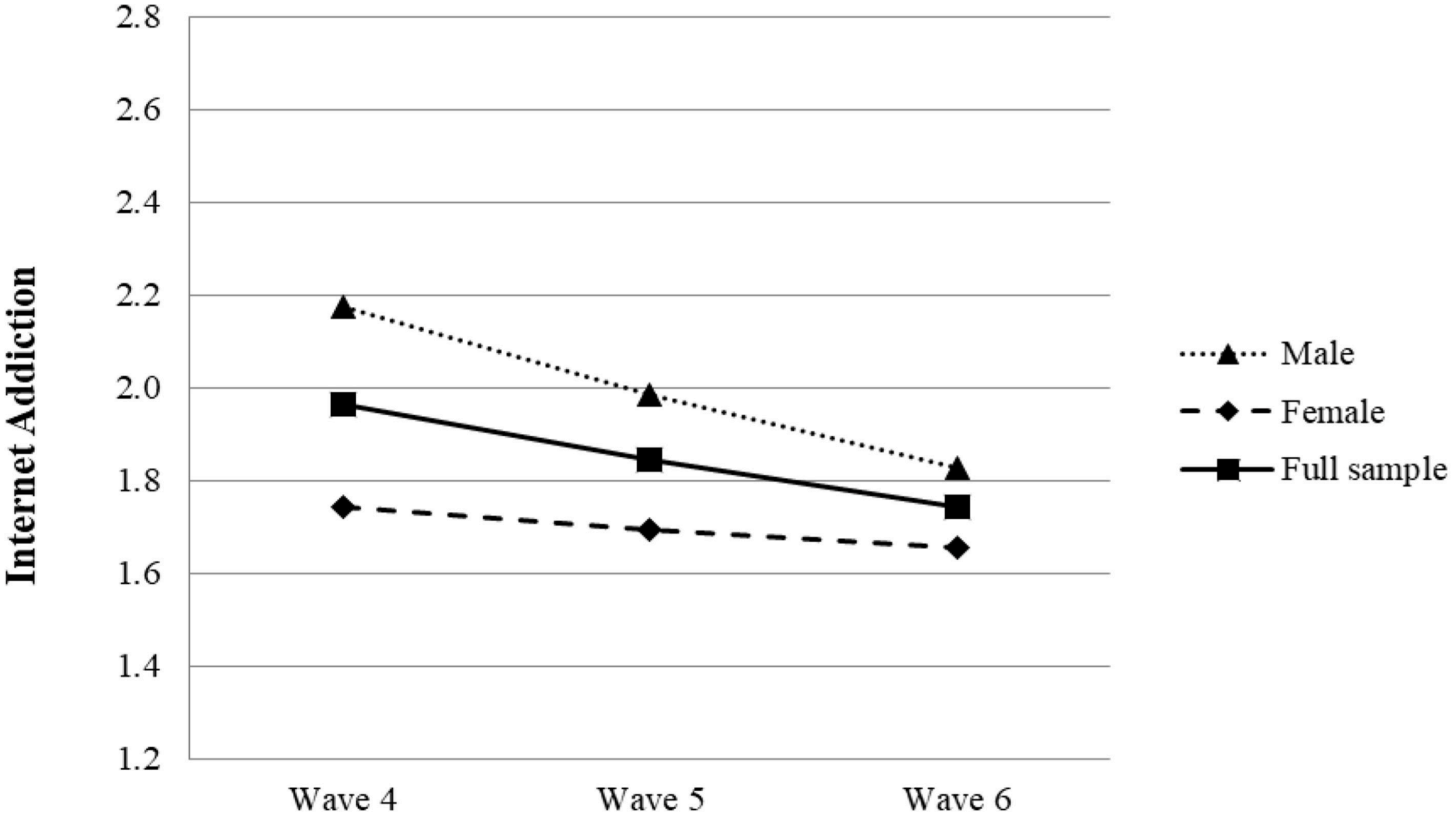
Growth trajectories of adolescent Internet addiction as a function of gender. The figure for the full sample was plotted based on Model 2 shown in Table 5. The figures for male and female samples were plotted based on results of gender-based analyses for Model 2 shown in Table 5.

Model 1 (i.e., the unconditional mean model) showed a relatively high intra-class correlation coefficient (ICC; 0.575) (see Table 5), indicating that individual differences accounted for 57.5% of the variance in IA levels. Thus, both Level 1 and Level 2 predictors should be considered (41). Comparison of model fit indices between Models 1 and 2 (i.e., the unconditional linear model) suggested that Model 2 fit the data better [Δχ${}_{\left(3\right)}^{2}$ = 81.85, *p* < 0.001, ΔAIC = 75.85, ΔBIC = 54.46]. According to Model 2, the level of adolescent IA declined slightly during the senior high school years (β = −0.120, *p* < 0.001) (see Figure 1).

**Table 5:** Results of IGC models (Model 1–3) for adolescent Internet addiction (Wave 4–6).

		**Model 1**		**Model 2**		**Model 3**		**Model 2 (Male)**		**Model 2 (Female)**	
		**Estimate**	**SE**	**Estimate**	**SE**	**Estimate**	**SE**	**Estimate**	**SE**	**Estimate**	**SE**
**FIXED EFFECTS**											
Intercept	β*_0*j*_*										
Intercept	γ*_00_*	1.851***	0.0341	1.965***	0.0395	2.192***	0.0852	2.176***	0.0609	1.743***	0.0492
Gender^a^	γ_01_					0.210***	0.0407				
Family economic status^b^	γ_02_					−0.221*	0.0878				
Family intactness^c^	γ_03_					−0.051	0.0573				
Linear Slope	β*_1_**j***										
Time	γ*_10_*			−0.1201***	0.0214	−0.164***	0.0465	−0.1902***	0.0329	−0.0469	0.0271
Gender^a^	γ_11_					−0.070**	0.0222				
Family economic status^b^	γ_12_					0.025	0.0480				
Family intactness^c^	γ_13_					0.030	0.0313				
**RANDOM EFFECTS**											
**Level 1 (within)**											
Residual	*r_*ij*_*	2.1250***	0.0383	1.8541***	0.0473	1.7818***	0.0472	2.2553***	0.0804	1.4335***	
**Level 2 (between)**											
Intercept	*u_0_**j***	2.8759***	0.0923	3.2028***	0.1291	3.1664***	0.1310	3.8946***	0.2194	2.3717***	
Time	*u_1*j*_*			0.3081***	0.0457	0.3415***	0.0466	0.3589***	0.0773	0.2450***	
**FIT STATISTICS**											
Deviance		38106.22		38024.38		35106.70		20277.69		17489.08	
AIC		38112.22		38036.38		35130.70		20289.69		17501.08	
BIC		38133.61		38079.15		35215.36		20328.45		17539.54	
Df		3		6		12		6		6	

*Model 1, unconditional mean model; Model 2, unconditional linear growth model; Model 3, conditional growth curve model (only with socio-demographic variables)*.

a *Female = −1, Male = 1*;

b *Having economic disadvantage = −1, Not having economic disadvantage = 1*;

c *Non-intact family = −1, Intact family = 1. AIC, Akaike Information Criterion; BIC, Bayesian Information Criterion*.

* *p < 0.05*;

** *p < 0.01*;

*** *p < 0.001*.

In Model 3, the control variables and time were treated as Level 2 predictors. Results showed that gender and family economic status were significant predictors of adolescents' IA level at Wave 4 (see Table 5). More specifically, male participants (β = 0.210, *p* < 0.001) or those with family economic disadvantage (β = −0.221, *p* < 0.05) had higher initial levels of IA. Additionally, gender had a significant effect on the rate of change in IA. Specifically, compared with female peers, male adolescents exhibited a faster drop in IA from Wave 4 to Wave 6 (β = −0.070, *p* < 0.01).

Further gender-based analyses for Model 2 revealed that the level of IA dropped significantly among male adolescents (β = −0.190, *p* < 0.001) and female adolescents, (β = −0.047, *p* = 0.04, one-tailed), although the magnitude was lower in female adolescents (see Table 5 and Figure 1). The results suggest that most of the variation in IA over time among the full sample is attributable to male adolescents.

### Predictive Effects of Parental Factors on the Initial Level of Adolescent IA

Model 4a, 4b, and 4c considered parental behavioral control, psychological control, and quality of the relationship between parents and children as predictors, respectively. Results of gender-based analyses for Model 4a, 4b, and 4c suggested that parental factors showed similar predictive effects on the initial level of IA among male and female adolescents. Thus, we only elaborated related findings below based on the full sample shown in Table 6.

**Table 6:** Results of IGC models with level-2 predictors for adolescent Internet addiction (Wave 4–6, full sample).

		**Model 4a**		**Model 4b**		**Model 4c**	
		**Estimate**	**SE**	**Estimate**	**SE**	**Estimate**	**SE**
**FIXED EFFECTS**							
Intercept	β*_0_**j***						
Intercept	γ_00_	2.121***	0.0846	2.198***	0.0845	2.132***	0.0846
Gender^a^	γ_01_	0.209***	0.0404	0.177**	0.0407	0.188***	0.0405
Family economic status^b^	γ_02_	−0.181*	0.0869	−0.224*	0.0871	−0.199*	0.0869
Family intactness^c^	γ_03_	0.005	0.0570	−0.050	0.0569	0.016	0.0573
Paternal behavioral control	γ_04_	−0.327***	0.0448				
Maternal behavioral control	γ_05_	−0.037	0.0446				
Paternal psychological control	γ_06_			0.073	0.0453		
Maternal psychological control	γ_07_			0.247***	0.0452		
Father–child relational quality	γ_08_					−0.258***	0.0446
Mother–child relational quality	γ_09_					−0.122**	0.0444
Linear Slope	β*_1_**j***						
Intercept	γ_10_	−0.143**	0.0466	−0.163***	0.0465	−0.151**	0.0466
Gender^a^	γ_11_	−0.068**	0.0223	−0.067*	0.0224	−0.062*	0.0223
Family economic status^b^	γ_12_	0.013	0.0479	0.025	0.0479	0.020	0.0479
Family intactness^c^	γ_13_	0.013	0.0314	0.028	0.0313	0.015	0.0316
Paternal behavioral control	γ_14_	0.082**	0.0247				
Maternal behavioral control	γ_15_	0.028	0.0246				
Paternal psychological control	γ_16_			0.018	0.0249		
Maternal psychological control	γ_17_			−0.052*	0.0249		
Father–child relational quality	γ_18_					0.038	0.0246
Mother–child relational quality	γ_19_					0.061*	0.0244
**RANDOM EFFECTS**							
**Level 1 (within)**							
Residual	*r_*ij*_*	1.7818***	0.0472	1.7818***	0.0472	1.7818***	0.0472
**Level 2 (between)**							
Intercept	*u_0*j*_*	3.0525***	0.1282	3.0857***	0.1290	3.0663***	0.1285
Time	*u_1*j*_*	0.3324***	0.0464	0.3393***	0.0465	0.3348***	0.0464
**FIT STATISTICS**							
Deviance		35036.06		35051.35		35042.44	
AIC		35068.06		35083.35		35074.44	
BIC		35180.93		35196.23		35187.32	
df		16		16		16	

a *Female = −1, Male = 1*;

b *Having economic disadvantage = −1, Not having economic disadvantage = 1*;

c *Non-intact family = −1, Intact family = 1. AIC, Akaike Information Criterion; BIC, Bayesian Information Criterion*.

* *p < 0.05*;

** *p < 0.01*;

*** *p < 0.001*.

For the full sample, compared to Model 3 shown in Table 5, Model 4a (Δχ${}_{\left(4\right)}^{2}$ = 70.64, *p* < 0.001, ΔAIC = 62.64, ΔBIC = 34.42), Model 4b (Δχ${}_{\left(4\right)}^{2}$ = 55.35, *p* < 0.001, ΔAIC = 47.35, ΔBIC = 19.13) and Model 4c (Δχ${}_{\left(4\right)}^{2}$ = 64.26, *p* < 0.001, ΔAIC = 56.26, ΔBIC = 28.04) had better model fits than Model 3 did (see Table 6).

According to Model 4a, while paternal behavioral control significantly predicted the initial level of adolescent IA at Wave 4 (β = −0.327, *p* < 0.001), maternal behavioral control did not (β = −0.037, *p* > 0.05). Thus, Hypothesis 1a was supported, while Hypothesis 1b was not.

According to Model 4b, while maternal psychological control was a significant predictor of the initial level of adolescent IA (β = 0.247, *p* < 0.001), paternal psychological control was not (β = 0.073, *p* > 0.05). These results did not support Hypothesis 1c, but they supported Hypothesis 1d.

According to Model 4c, the quality of the relationship between fathers and children (β = −0.258, *p* < 0.001) and between mothers and children (β = −0.122, *p* < 0.01) were both significant predictors of children's level of IA at Wave 4. Thus, Hypotheses 1e and 1f were supported.

### Predictive Effects of Parental Factors on the Growth Rate of Adolescent IA

It was noteworthy that parental factors generally did not have significant impacts on the change rate of female adolescents' IA. Predictive effects of parental factors on the growth rate of IA based on the full sample were largely contributed by male adolescents (i.e., parental factors showed a similar pattern of predictive effects between the full sample and the male sample). In the following sections, we mainly outline the results based on the full sample (see Table 6).

**Figure 2:**
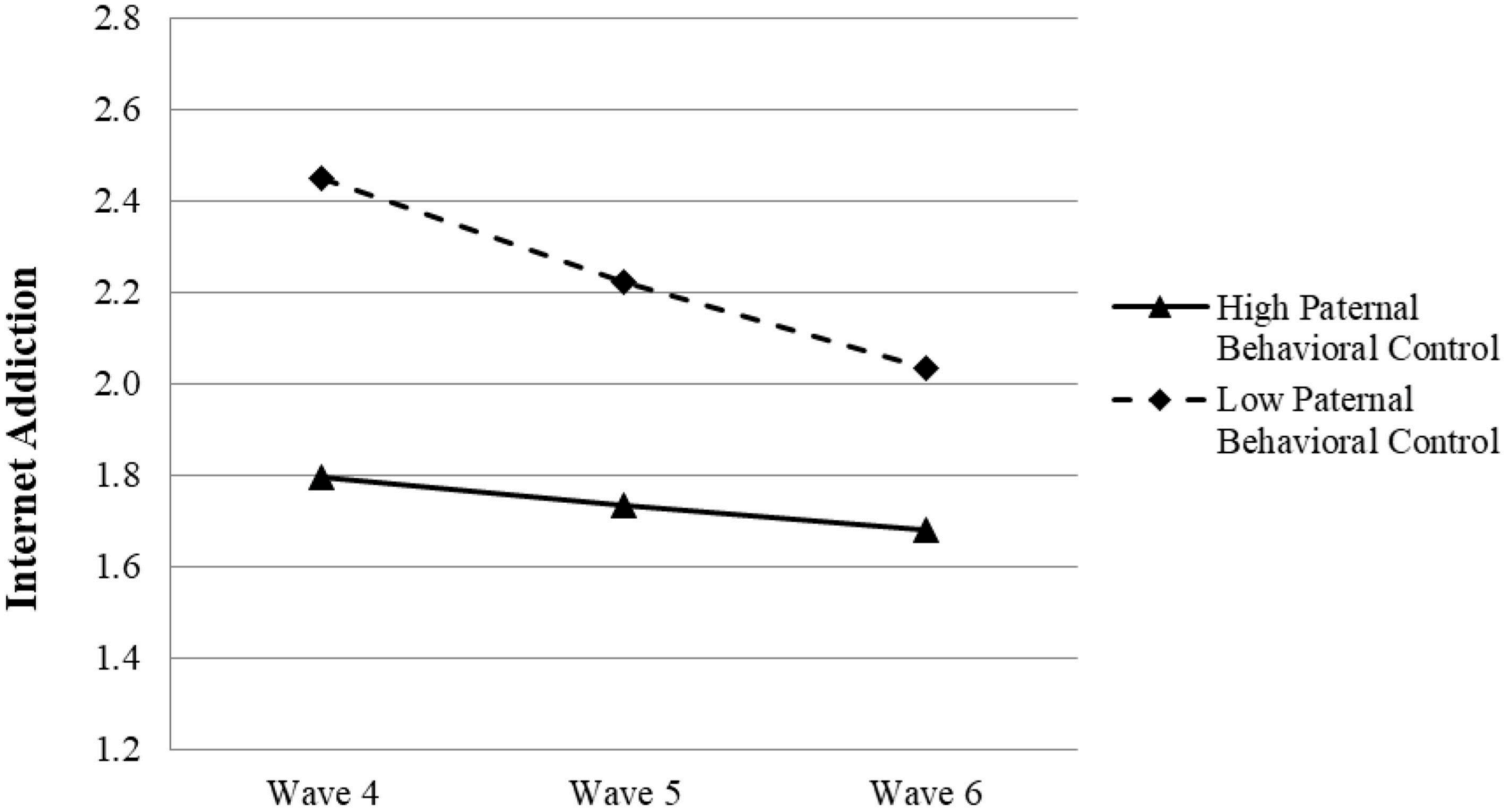
Growth trajectories of adolescent Internet addiction as a function of paternal behavioral control. The figures were plotted based on Model 4a shown in Table 6. High level indicates 1SD higher than the mean value; low level indicates 1SD lower than the mean value.

It was found that paternal behavioral control significantly predicted the growth rate of children's IA across three waves (β = 0.082, *p* < 0.01), but maternal behavioral control did not (β = 0.028, *p* > 0.05) (see Table 6). Results indicated that participants with higher paternal behavioral control showed a lower level of IA at Wave 4 but a slower decrease in IA over time (see Figure 2). Although parental behavioral control tended to significantly predict the linear change rate of adolescent IA, the direction was contrary to our hypothesis. Thus, both Hypotheses 2a and 2b were not supported.

**Figure 3:**
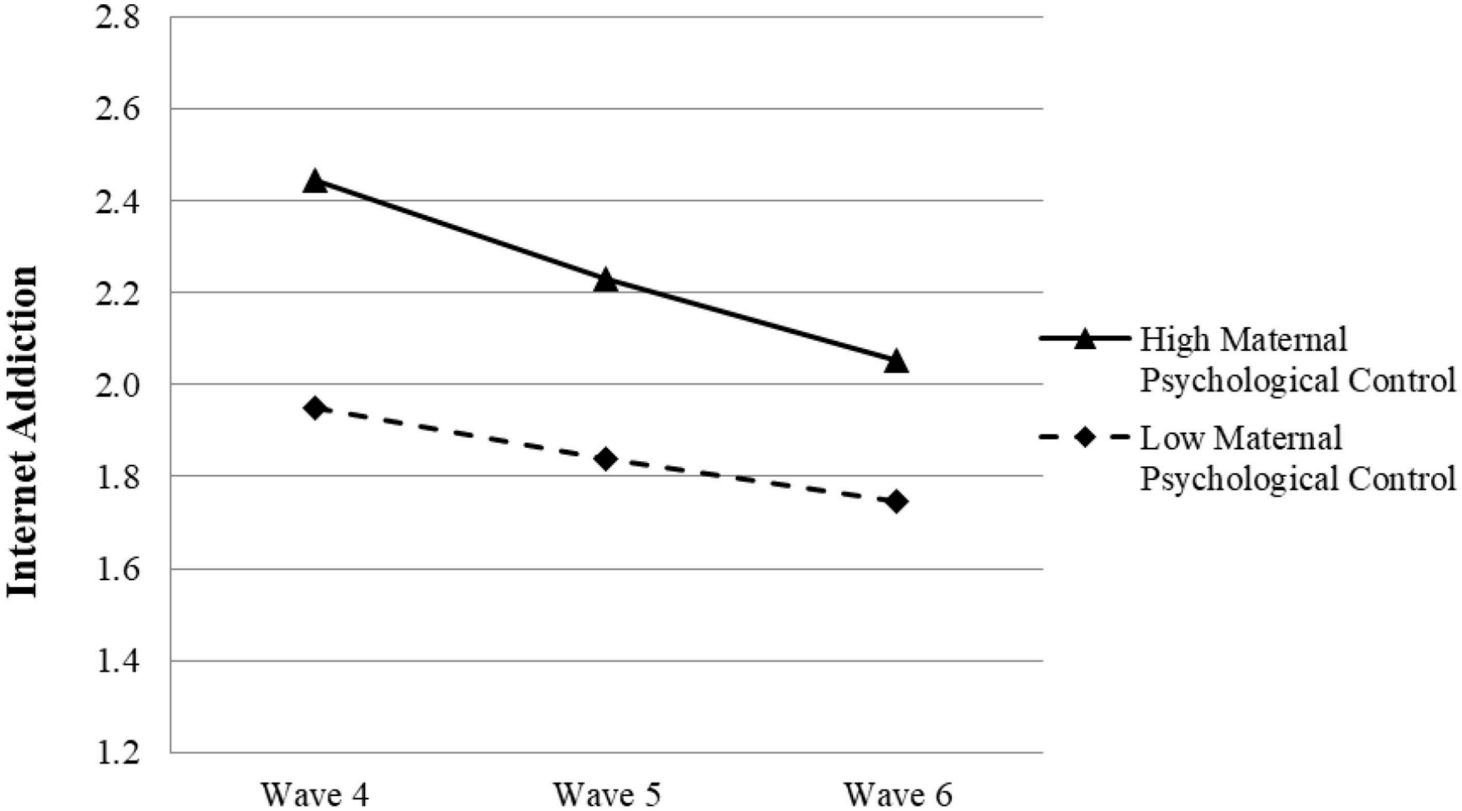
Growth trajectories of adolescent Internet addiction as a function of maternal psychological control. The figures were plotted based on Model 4b shown in Table 6. High level indicates 1SD higher than the mean value; low level indicates 1SD lower than the mean value.

Based on the results presented in Table 6, while paternal psychological control was not significantly associated with the growth rate of adolescent IA (β = 0.018, *p* > 0.05), higher maternal psychological control was linked to a faster decrease in children's IA from Wave 4 to Wave 6 (β = −0.052, *p* < 0.05, see Figure 3). The direction of maternal psychological control's predictive effect on the change rate in adolescent IA was opposite to our hypothesis. Hence, Hypotheses 2e and 2f were not supported.

**Figure 4:**
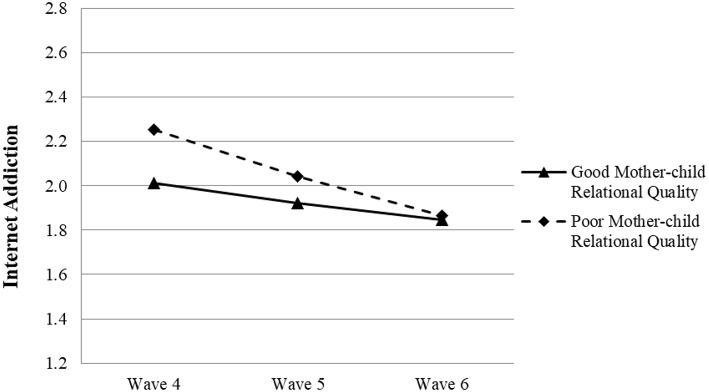
Growth trajectories of adolescent Internet addiction as a function of mother–child relational quality. The figures were plotted based on Model 4c shown in Table 6. Good quality indicates 1SD higher than the mean value; poor quality indicates 1SD lower than the mean value.

With reference to Model 4c, the quality of relationship between mothers and children was a significant predictor of the growth rate of adolescent IA (β = 0.061, *p* < 0.05), while father–child relationship quality was not (β = 0.038, *p* > 0.05) (see Table 6). These findings suggested that a better mother–child relationship predicted a slower decline in IA during senior high school years (see Figure 4). Thus, Hypothesis 2c and 2d were not supported.

### Relative Concurrent and Longitudinal Influence of Paternal and Maternal Factors

Results of the relative concurrent and longitudinal predictive effects of paternal and maternal factors have been present in Tables 7, 8. Further analyses, including interactions between gender and each parental factor, revealed that children's gender did not substantially moderate the concurrent and longitudinal predictive effects of parental factors. In this case, we outlined regression results based on the full sample in sections below.

**Table 7:** Concurrent predicting effects of parent–child subsystem qualities on Internet addiction.

**Model**	**Predictors**	**Wave 4 Internet addiction**^a^			**Wave 5 Internet addiction**^b^			**Wave 6 Internet addiction**^c^		
		**β**	***t***	**Cohen's *f^**2**^***	**β**	***t***	**Cohen's *f^**2**^***	**β**	***t***	**Cohen's *f^**2**^***
1	Gender^d^	0.10	5.525***	0.011	0.05	2.43*	0.002	0.05	2.48*	0.002
	FES^e^	−0.04	−2.21*	0.002	−0.05	−2.76**	0.003	−0.03	−1.64	0.001
	FI^f^	−0.02	−0.77	0.000	−0.01	−0.60	0.000	0.00	0.19	0.000
	*R^2^* change	0.013	0.005	0.003
	*F* change	12.08***	5.00**	2.879*
2	PBC	−0.16	−6.14***	0.013	−0.15	−5.89***	0.012	−0.11	−4.39***	0.007
	PPC	0.10	4.94***	0.009	0.14	7.09***	0.018	0.13	6.40***	0.014
	FCRQ	−0.02	−0.56	0.000	0.03	1.15	0.000	0.02	0.80	0.000
	*R^2^* change	0.034	0.029	0.023
	*F* change	36.75***	28.87***	21.43***
3	MBC	−0.07	−2.83**	0.003	−0.04	−1.42	0.001	−0.03	−1.31	0.001
	MPC	0.13	6.25***	0.014	0.12	5.89***	0.012	0.13	6.48***	0.015
	MCRQ	−0.02	−0.92	0.000	−0.02	−0.69	0.000	0.00	0.04	0.000
	*R^2^* change	0.024	0.016	0.018
	*F* change	23.21***	15.87***	17.18***
4	PBC	−0.15	−5.53***	0.011	−0.15	−5.74***	0.012	−0.12	−4.16***	0.006
	PPC	0.05	2.37*	0.002	0.11	4.99***	0.009	0.09	3.81***	0.005
	FCRQ	−0.01	−0.35	0.000	0.04	1.33	0.001	0.02	0.83	0.000
	MBC	−0.01	−0.34	0.000	0.03	1.03	0.000	0.02	0.63	0.000
	MPC	0.10	4.57***	0.007	0.07	3.23**	0.004	0.09	4.08***	0.006
	MCRQ	−0.02	−0.66	0.000	−0.03	−1.08	0.000	−0.01	−0.28	0.000
	*R^2^* change	0.044			0.035			0.029		
	*F* change	22.00***			17.49***			14.32***		

*For Model 2–4, social-demographic variables were controlled*.

a *Parent–child subsystem qualities measured at Wave 4 were used*;

b Parent–child subsystem qualities measured at Wave 5 were used;

c *Parent–child subsystem qualities measured at Wave 6 were used*;

d *Female = −1, Male = 1*;

e *Having economic disadvantage = −1, Not having economic disadvantage = 1*;

f *Non-intact family = −1, Intact family = 1. FES, Family economic status; FI, Family intactness; PBC, Paternal behavioral control; PPC, Paternal psychological control; FCRQ, Father–child relational quality; MBC, Maternal behavioral control; MPC, Maternal psychological control; MCRQ, Mother–child relational quality*.

* *p < 0.05*;

** *p < 0.01*;

*** *p < 0.001*.

**Table 8:** Longitudinal predicting effects of parent–child subsystem qualities on Internet addiction.

**Model**	**Predictors**	**Wave 5 Internet addiction**			**Wave 6 Internet addiction**		
		**β**	***t***	**Cohen's *f^**2**^***	**β**	***t***	**Cohen's *f^**2**^***
1	Gender^a^	0.05	2.43*	0.002	0.05	2.48*	0.002
	FES^b^	−0.05	−2.76**	0.003	−0.03	−1.64	0.001
	FI^c^	−0.01	−0.60	0.000	0.004	0.19	0.000
	*R^2^* change		0.005			0.003	
	*F* change		5.00**			2.88*	
2	PBC	−0.09	−3.53***	0.004	−0.08	−2.96**	0.003
	PPC	0.07	3.56***	0.004	0.09	4.29***	0.006
	FCRQ	−0.03	−1.19	0.000	−0.02	−0.70	0.000
	*R^2^* change		0.017			0.015	
	*F* change		16.88***			14.09***	
3	MBC	−0.06	−2.47*	0.002	−0.04	−1.51	0.001
	MPC	0.10	5.10***	0.009	0.10	4.82***	0.008
	MCRQ	0.01	0.35	0.000	0.01	0.42	0.000
	*R^2^* change		0.013			0.010	
	*F* change		12.13***			9.22***	
4	PBC	−0.08	−2.97**	0.003	−0.07	−2.72**	0.003
	PPC	0.03	1.43	0.001	0.05	2.39*	0.002
	FCRQ	−0.04	−1.38	0.001	−0.03	−0.89	0.000
	MBC	−0.02	−0.90	0.000	0.00	−0.09	0.000
	MPC	0.09	3.88***	0.005	0.07	3.19**	0.004
	MCRQ	0.02	0.79	0.000	0.02	0.60	0.000
	*R^2^* change		0.023			0.018	
	*F* change		11.14***			8.93***	

*For Model 2–4, social-demographic variables were controlled; parent–child subsystem qualities measured at Wave 4 were used as predictors*;

a *Female = −1, Male = 1*;

b Having economic disadvantage = −1, Not having economic disadvantage = 1;

c *Non-intact family = −1, Intact family = 1. FES, Family economic status; FI, Family intactness; PBC, Paternal behavioral control; PPC, Paternal psychological control; FCRQ, Father–child relational quality; MBC, Maternal behavioral control; MPC, Maternal psychological control; MCRQ, Mother–child relational quality*.

* *p < 0.05*;

** *p < 0.01*;

*** *p < 0.001*.

First, after the three social-demographic factors were controlled, the three concurrent father-related factors explained 3.4, 2.9, and 2.3% of variance in children's IA at Wave 4, 5, and 6 respectively. Table 7 shows that father–child relationship quality did not show cross-sectional effect in any wave. While paternal behavioral control significantly predicted children's IA negatively (Wave 4: β = −0.16, *p* < 0.001, Cohen's *f*^2^ = 0.013; Wave 5: β = −0.15, *p* < 0.001, Cohen's *f*^2^ = 0.012; Wave 6: β = −0.11, *p* < 0.001, Cohen's *f*^2^ = 0.007). Paternal psychological control positively predicted children's IA at the three time points (Wave 4: β = 0.10, *p* < 0.001, Cohen's *f*^2^ = 0.009; Wave 5: β = 0.14, *p* < 0.001, Cohen's *f*^2^ = 0.018; Wave 6: β = 0.13, *p* < 0.001, Cohen's *f*^2^ = 0.014). For longitudinal effects, paternal factors assessed at Wave 4 explained 1.7 and 1.5% of the variance in children's IA at the latter two time points, respectively. Table 8 shows that the father–child relationship did not predict adolescent IA over time. While paternal behavioral control was a negative longitudinal predictor of children's IA (Wave 5: β = −0.09, *p* < 0.001, Cohen's *f*^2^ = 0.004; Wave 6: β = −0.08, *p* < 0.01, Cohen's *f*^2^ = 0.003), paternal psychological control was a positive longitudinal predictor of children's IA (Wave 5: β = 0.07, *p* < 0.001, Cohen's *f*^2^ = 0.004; Wave 6: β = 0.09 *p* < 0.001, Cohen's *f*^2^ = 0.006).

Second, the three concurrent maternal factors explained 2.4, 1.6, and 1.8% of the variance in children's IA across Wave 4 to 6 (see Table 7), respectively. Specifically, the quality of the relationship between mothers and children was not a significant concurrent predictor of children's IA. Maternal behavioral control had a significant negative predictive effect at Wave 4 (β = −0.07 *p* < 0.01, Cohen's *f*^2^ = 0.003), but not at Waves 5 and 6. In contrast, maternal psychological control positively predicted children's IA (β ranged between 0.12 and 0.13, *ps* < 0.001, Cohen's *f*^2^ ranged between 0.012 and 0.015). The longitudinal effects of maternal factors measured at Wave 4 explained 1.3 and 1.0% of the variance in children's IA at the latter two waves, respectively. Table 8 shows that mother–child relational quality did not predict children's IA over time. In contrast, the longitudinal effect of maternal behavioral control on adolescent IA was significant at Wave 5 (β = −0.06, *p* < 0.05, Cohen's *f*^2^ = 0.002), but not significant at Wave 6 (β = −0.04, *p* > 0.05, Cohen's *f*^2^ = 0.001). However, maternal psychological control predicted children's IA at the two waves (Wave 5: β = 0.10, *p* < 0.001, Cohen's *f*^2^ = 0.009; Wave 6: β = 0.10, *p* < 0.001, Cohen's *f*^2^ = 0.008).

The above results indicated that paternal factors explained a larger amount of variance in adolescent IA than did maternal factors. Thus, the findings tended to support Hypothesis 3a rather than Hypothesis 3b.

Third, when the concurrent paternal factors and maternal factors were investigated simultaneously, they uniquely explained 4.4, 3.5, and 2.9% of the variance in children's IA at the three waves, respectively (see Table 7). Specifically, while father– and mother–child relationship quality and maternal behavioral control did not show cross-sectional effects, paternal behavioral control (β ranged between −0.15 and −0.12, *ps* < 0.001, Cohen's *f*^2^ ranged between 0.006 and 0.011) and psychological control (β ranged between 0.05 and 0.11, *ps* < 0.05, Cohen's *f*^2^ ranged between 0.002 and 0.009), as well as maternal psychological control (β ranged between 0.07 and 0.10, *ps* < 0.01, Cohen's *f*^2^ ranged between 0.004 and 0.007) were significant concurrent predictors in the three waves. Regarding longitudinal effects, parental factors measured at Wave 4 explained 2.3% and 1.8% of the variance in children's IA measured at the latter two waves, respectively (see Table 8). Among the six parental factors, only paternal behavioral control and maternal psychological control showed robust longitudinal effects on children's IA. While paternal behavioral control exerted negative effects on children's IA over time (Wave 5: β = −0.08, *p* < 0.01, Cohen's *f*^2^ = 0.003; Wave 6: β = −0.07, *p* < 0.01, Cohen's *f*^2^ = 0.003), maternal psychological control showed positive longitudinal effects (Wave 5: β = 0.09, *p* < 0.001, Cohen's *f*^2^ = 0.005; Wave 6: β = 0.07, *p* < 0.01, Cohen's *f*^2^ = 0.004).

The present findings are summarized in Table 1 with reference to the hypotheses.

## Discussion

The present study examined parental influence on the development of Internet Addiction (IA) among Chinese adolescents. Three research questions were focused upon in this study. First, by separating paternal and maternal factors, our study examined the impacts of multiple parenting factors and quality of the relationship between parents and children on the initial level of children's IA. Second, the predictive effects of parental factors on the growth rate of children's IA were also investigated. Third, we also examined and compared the influence of different parental factors on adolescent IA at a single time point and over time. While a recent study has addressed these questions in early adolescents (28), the present study involved late adolescents in senior high school. In addition, gender-based analyses were conducted to further explore the effects of children's gender.

While most of the present findings are similar to the observations reported on early adolescents, some are different from these previous findings (28). For the first research question, male adolescents showed a higher initial level of IA than did females, which is in line with previous findings (28, 43). In addition, we observed a decline trend in adolescent IA. It is plausible that students in the higher grade will be less devoted to activities related to the Internet as they must prepare for the university entrance examination. Additionally, the magnitude of decline in IA was greater among male adolescents than that among female students. This gender difference may be attributed to female adolescents' relatively low level of initial IA, which was unlikely to decline substantially (i.e., floor effect). Furthermore, we did not observe a significant effect regarding children's gender and parental impacts on IA levels, which is inconsistent with the previous findings that parental psychological control was more influential on girls' than on boys' externalizing behaviors (26, 30). Nevertheless, as no previous research has tested gender effect regarding change in IA over time, more research is needed to portray a conclusive picture.

Regarding the parental influence on the initial level of IA that was addressed by the first research question, four hypotheses (1a, 1d, 1e, and 1f) were supported, whereas Hypotheses 1b and 1c were not. Specifically, when each aspect of the quality of the parent–child subsystem was examined separately in individual growth curve (IGC) models based on the full sample, higher paternal but not maternal behavioral control, and better relationships with both parents, were significantly associated with lower levels of initial adolescent IA. Additionally, higher maternal but not paternal psychological control predicted a higher level of initial adolescent IA. Gender-based IGC models showed similar findings for male and female adolescents. On the one hand, these findings suggested that parents' behavioral control and better parent-child relationships are positive parenting factors, whereas psychological control is a negative factor, regardless of children's gender. This conclusion echoes the previous findings observed in both Chinese and Western contexts (7, 28, 44).

On the other hand, the observations shed light on the differential influence of fathers' and mothers' factors. Specifically, paternal parenting had a closer association with adolescent IA via behavioral control, while mothers exerted stronger influence through psychological control. These findings seem to be different from the results reported in Shek et al.'s (28) research, which found that paternal and maternal factors tend to function in the same way in predicting the initial level of adolescent IA in early adolescence. However, as indicated by the estimated coefficients of predictors in Shek et al.'s (28) research, paternal behavioral control in comparison to maternal behavioral control, and maternal psychological control in comparison to paternal psychological control, tended to have a stronger association with the initial level of adolescent IA. Taken together, these findings suggest that fathers and mothers might contribute to the development of adolescent IA through different parental behaviors at different stages of adolescence. Such an assumption can help explain the existing mixed findings on paternal vs. maternal impacts on adolescent development. This will be discussed with reference to regression analyses below.

For the second research question, all the hypotheses based on general theoretical models were not supported. However, the findings are consistent with the previous observations among early adolescents (28). It was found that higher paternal behavioral control and better mother–child relationship predicted a slower decline in adolescent IA during senior high school years. Meanwhile, higher psychological control was linked with a faster decrease in adolescent IA. In short, these findings suggest that—similar to the situation in early adolescence—parental impacts also gradually diminish over time during late adolescence. These observations coincide with the decreasing amount of variance in adolescent IA explained by parental factors, as revealed by regression analyses. For example, the percentage of adolescent IA accounted by all concurrent parental factors decreased from 4.4% at Wave 4 to 2.9% at Wave 6. As suggested by Shek et al. (28), this finding may be attributable to adolescents' decreasing dependence on parents and increasing devotion to other types of social relationships (e.g., peer relationships). Additionally, at later stages of senior high school, all students in Hong Kong must invest most of their time and energy into preparing for the public examination. Thus, adolescents would generally be less different from each other in terms of their IA behaviors. Furthermore, those with IA problems may have dropped out from the study. Therefore, parental influence on adolescent IA may become less significant. Nevertheless, as the present study is a pioneering attempt, these findings need to be confirmed in future replication studies.

Regarding the contribution of fathers and mothers to children's IA levels (i.e., the third research question), the present study revealed that paternal factors accounted for a higher percentage of adolescent IA than did maternal factors, both concurrently and longitudinally. The general greater paternal impact vs. maternal impact on children's IA is in line with previous studies involving different developmental outcome measures. For example, using global treatment style, responsiveness, and demandingness to index parenting qualities, Shek (45, 46) reported that, relative to maternal parenting qualities, paternal parenting qualities had a greater impact on Chinese adolescent morbidities such as hopelessness and psychological well-being indexed by self-esteem, purpose of life, and life satisfaction. One recent study also identified that paternal, but not maternal, expectations regarding children's future significantly affected children's well-being (47). Generally speaking, fathers are less involved in parenting tasks than mothers are, especially in Chinese society (48). In the present study, fathers were less controlling and had a poorer relationship with the children as compared to mothers. However, as fathers occupy a superior and controlling role in Chinese families, fathers may be more influential in their children's developmental outcomes although they are less involved and more detached. This interpretation is consistent with other findings in early adolescents (49).

Different from the present study, Shek et al. (28) found that paternal and maternal factors had equal impacts on children's IA levels. One possible explanation is that the age of the adolescents may have served as a moderator. Specifically, Shek et al.'s (28) study involved early adolescents (mean age = 12.59 years old) who were younger as compared to the present participants. A recent study in Korea supported the potential moderating effect of children's age on parental impacts (50). The study showed that, while parental affection negatively predicted children's problematic mobile game use in the elementary school group, parental monitoring lead to less problematic mobile game use in the high school group. Future research could directly compare paternal impacts on adolescent IA with reference to different age groups to test the possibility that paternal factors might have generally greater impacts as compared to maternal factors among older adolescents.

Differential parental impacts should also be discussed with reference to specific aspects of parenting. As mentioned earlier, when each dimension of the parent–child subsystem was considered in the IGC models, paternal behavioral control but not maternal behavioral control, and maternal psychological control but not paternal psychological control, were significantly linked with the initial level of children's IA. Basically, these findings are consistent with the concurrent and longitudinal predictive effects derived from the regression analyses. When paternal and maternal factors were examined simultaneously in regressions, paternal behavioral control and maternal psychological control were the two most robust predictors of adolescent IA, both concurrently and longitudinally. The consistent findings obtained from different analytical approaches informed the reliability of these findings.

Based on the findings, it can be conjectured that the impact of paternal factors is greater in terms of behavioral control, while maternal parenting is more influential in terms of psychological control over time. In previous studies, while some findings suggested stronger paternal impacts on adolescent development, some others supported stronger maternal impacts. One possible explanation for the mixed findings is that most of these studies focused on overall parenting without considering different dimensions of parenting or only referred to one dimension of parenting. For example, in studies that identified greater impacts of maternal psychological control on adolescent development outcomes, behavioral control or other parental factors were not examined simultaneously (25, 51). Chen et al. (52) identified stronger influence of fathers' indulgence, but they did not take psychological control into account. In fact, the results in a few studies that considered different domain-specific parental factors showed that both parents' influence differed from each other on specific aspects. For example, while maternal control showed a greater impact on children's achievement motivation and positive youth development than did paternal control, paternal sacrifice exerted a stronger influence than did maternal sacrifice on adolescents' positive youth development (53, 54). Thus, our findings suggest that it is important to distinguish between different processes of parenting in examining and comparing paternal and maternal influences.

There are two additional observations which deserve attention. First, the concurrent negative impacts of both parents' psychological control on adolescent IA are consistent with findings on early adolescence (28). Researchers advocated that parental psychological control would ruin children's age-appropriate sense of autonomy, which in turn results in adolescent misbehaviors and damaged psychological well-being. However, most of the findings were derived from samples of adolescents in Western cultures, which value autonomy and independence. Some scholars argued that in Asian countries, including China, parental psychological control may not be so detrimental as cultural norms in these areas emphasize children's obligations to the family and interdependence rather than independence (8, 55). Nevertheless, recent studies showed that in Chinese adolescents, parents' psychological control was concurrently associated with children's maladjustment, such as depression and anxiety as well as hopelessness (56). Thus, the present study adds to the extant literature by showing that the negative impacts of parents' psychological controlling behavior may be universal.

Second, the present longitudinal findings suggest that, during senior high school years, maternal psychological control might be more influential than paternal psychological control is. This observation is not consistent with the general view that negative paternal parenting is especially detrimental due to the greater authority and power of fathers in the family and the related attributes of the father–child dyad (30). For example, paternal psychological control was more closely associated with early adolescents' externalizing behaviors over time than maternal psychological control was (26, 30). Likewise, in early adolescence, paternal psychological control served as a more robust longitudinal predictor than maternal psychological control did for adolescent IA (28). Among university students, paternal, but not maternal, negative parenting in terms of denying and overprotectiveness resulted in a higher level of children's problematic Internet use (57). Nevertheless, it is possible that paternal and maternal negative parenting impact different adolescent developmental outcomes. For instance, Shek (58) found that paternal—but not maternal—psychological control showed a significant predictive effect on adolescents' life satisfaction in 1 year. Meanwhile, maternal—but not paternal—psychological control was a significant predictor of changes in adolescents' self-esteem. The present findings suggest the importance of examining this issue with reference to different measures of development outcomes among different age groups of adolescents.

The present study sheds light on the differential parental impacts on the concurrent and future levels and the growth rate of adolescents' IA during senior high school years. However, several limitations should be noted. First, the present study employed a quantitative design, which was unable to reveal the subjective feelings of participants and delineate potential mechanisms behind the quantitative findings. To portray a comprehensive picture and to triangulate the present findings, it is necessary to further investigate differential parental impacts using qualitative research strategies, such as focus group interview and case study.

Second, the utilized data were obtained only from adolescent participants' self-report. Although social desirability is a major shortcoming of self-report methodology, self-report data have commonly been collected in adolescent research, especially in longitudinal studies [e.g., (59)], possibly due to ethical and practical concerns. Besides, it is efficient and cost-effective, and it can be argued that adolescents themselves know their own experiences and lives much better than others. In the present study, to reduce social desirability bias as much as possible, anonymity was emphasized, and the participants were clearly instructed to provide answers based on their true perceptions without communicating with others. Nevertheless, future research could employ individual interviews in addition to self-report or involve different informants such as parents and teachers.

Third, the present study surveyed Chinese adolescents in a single region (i.e., Hong Kong). To verify and expand the generalizability of the present findings, future studies will benefit from investigating related questions among adolescents in other Chinese communities, such as those from mainland China, and adolescents from other ethnicities. Fourth, from Wave 4 to Wave 6, 899 participants withdrew from the study. In particular, more than 500 students withdrew from the study after Wave 4. This fact might affect the present findings. However, comparisons between the matched sample and dropouts suggested that there were no substantial differences in key variables between the two groups. Thus, the present findings were unlikely to be significantly affected by systematic attrition. Finally, as a control variable, family intactness was indicated by parental marital status. Since there were other factors related to family intactness, such as family members' residential status (e.g., whether children are living with their parents) (60), future studies could consider more comprehensive measures of family intactness.

## Data Availability

The datasets generated for this study are available on request to the corresponding author.

## Author Contributions

DS designed the project and contributed to all steps of the work. XZ contributed to the development of the idea and data interpretation. XZ and DD drafted the work and revised it based on the critical comments and editing provided by DS. All authors approve of the final version of the manuscript and agree to be accountable for all aspects of the work in ensuring that questions related to the accuracy or integrity of any part of the work are appropriately investigated and resolved.

### Conflict of Interest Statement

The authors declare that the research was conducted in the absence of any commercial or financial relationships that could be construed as a potential conflict of interest.
